# Beyond the Individual: An Ecological Analysis of Workplace Bullying in Aotearoa New Zealand Midwifery

**DOI:** 10.1155/jonm/1679262

**Published:** 2026-10-18

**Authors:** Vasudha Rao, Beth Tootell

**Affiliations:** ^1^ School of Management and Marketing Massey University Turitea New Zealand massey.ac.nz

## Abstract

**Aim:**

This study examined how midwives described workplace bullying and the individual, relational, organisational and wider professional or policy conditions associated with their experiences.

**Background:**

This exploratory qualitative descriptive study comprised Phase II of a two‐phase investigation of workplace bullying among midwives in Aotearoa New Zealand.

**Methods:**

Seven practising midwives from varied career stages and practice contexts participated in semistructured online interviews. Two researchers independently coded the transcripts, compared their coding and collaboratively reviewed developing patterns and themes. Data‐led analysis generated four process‐oriented themes, which were then interpreted using Johnson’s Ecological Model of Workplace Bullying.

**Results:**

Participants described public belittling, professional undermining, exclusion, fear, psychological distress, diminished professional confidence and withdrawal. Four themes were constructed: being diminished and pushed out, hierarchy turns vulnerability into control, organisational silence protects harmful practice and system conditions normalise and isolate. Participants connected these experiences with relational and professional hierarchies, reporting barriers, perceived leadership inaction, workforce pressures, employment arrangements and professional or cultural norms. Cross‐level analysis suggested possible connections between interpersonal devaluation, organisational responses and wider workforce and professional conditions.

**Conclusions:**

The findings suggest that workplace bullying may extend beyond interpersonal conflict and be sustained through interacting relational, organisational and structural influences. This contextually grounded study identifies potential intervention points across ecological levels without claiming prevalence, causation or effects on patient safety across the wider midwifery workforce. Larger and more diverse studies incorporating organisational and regulatory perspectives are needed to examine these pathways and evaluate multilevel interventions.

**Implications for Nursing Management:**

Responses should extend beyond individual coping and support to address relational conduct, organisational accountability and wider professional conditions. For nursing management, recognising bullying as more than an interpersonal problem requires considering how leadership responses, reporting processes, workforce pressures, employment arrangements and professional hierarchies may shape workplace experiences. These remain candidate areas for further evaluation.

## 1. Introduction

Workplace bullying, understood as repeated and persistent negative acts that create a hostile work environment [1], is pervasive across healthcare, with consequences for staff well‐being, organisational functioning and patient care. Midwives may be particularly vulnerable because their relational, autonomous and high‐stress work involves continual interaction with patients, multidisciplinary teams and hierarchical systems [2–4]. International research identifies colleagues, managers and allied health professionals as perpetrators and links bullying among midwives to job dissatisfaction, psychological harm and workforce attrition [4, 5].

Although concern about bullying in Aotearoa New Zealand maternity services is increasing, research has largely focused on individual and interpersonal factors, with limited attention to organisational and systemic influences. Aotearoa New Zealand‐specific qualitative evidence concerning midwifery remains scarce, despite research indicating that bullied midwives may leave the profession [6]. This gap is particularly important amid workforce shortages and resource pressures and highlights the need for contextually grounded, systems‐based research.

This study reports Phase II of a two‐phase study of workplace bullying among midwives in Aotearoa New Zealand. Phase I used a mixed‐methods design combining the Negative Acts Questionnaire‐Revised (NAQ‐R) with open‐ended responses to examine the prevalence and broad characteristics of bullying nationwide [7]. The qualitative responses provided preliminary insight into its patterns, contexts and perceived drivers. Concerning levels of mistreatment indicated the need to investigate participants’ experiences in greater depth. Phase I identified bullying associated with authority figures, exclusion, false narratives and victim blaming and fear of speaking up. It also identified reported consequences for mental health, workforce turnover and student learning. These findings informed broad Phase II interview domains concerning power and context, exclusion and professional undermining, reporting and voice and personal and professional consequences.

Phase II therefore used qualitative interviews and Johnson’s [8] Ecological Model of Workplace Bullying (Figure 1) to examine how intertwined influences shaped bullying across four interconnected levels. The microlevel concerns individual characteristics, coping, vulnerability and power; the mesolevel encompasses collegial and supervisory relationships, communication and interpersonal power; the exolevel concerns organisational structures and cultures, including leadership, workload, staffing and institutional norms; and the macro level captures wider professional, regulatory and sociocultural influences, including gendered norms, professional hierarchies and accountability mechanisms. The framework is particularly relevant to midwifery, where professional hierarchies, interprofessional boundaries and policy environments intersect.

**FIGURE 1:**
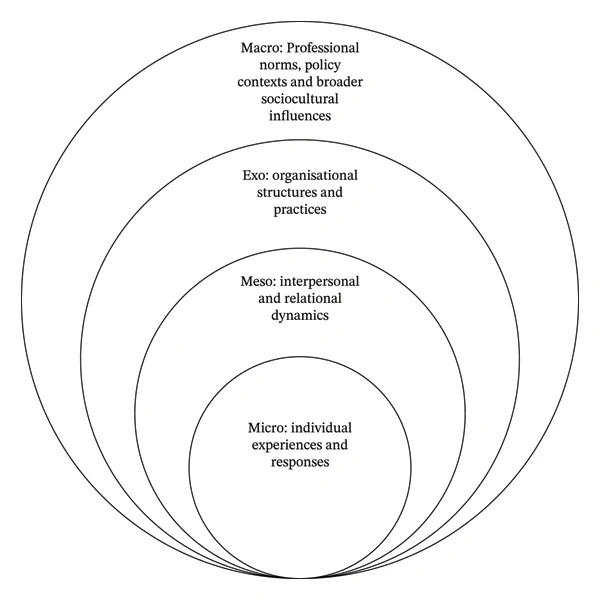
Author‐created representation of the ecological levels informing the analysis, based on Johnson’s [8] ecological model of workplace bullying.

Accordingly, this exploratory qualitative study aimed to examine how midwives described workplace bullying and the perceived influences that shaped and sustained it across individual, relational, organisational and wider professional or policy contexts. It asked: How do participating midwives describe the conditions associated with workplace bullying across the levels of Johnson’s ecological model?

### 1.1. Literature

Workplace bullying is commonly understood as repeated behaviour that undermines, isolates or interferes with an individual’s capacity to work [9]. It may include persistent criticism, sarcasm, exclusion, professional marginalisation and overt hostility, and is often discussed alongside harassment, incivility and horizontal or vertical violence [10–12]. These behaviours may intersect with discrimination and structural inequality; internationally qualified health professionals, for example, may experience mistreatment associated with accent, ethnicity or the perceived legitimacy of their qualifications [13].

Healthcare environments are recognised as high‐risk settings because professional hierarchies, high workloads and interdependent clinical work can intensify both exposure to bullying and its consequences [3, 14, 15]. Bullying has been associated with anxiety, depression, burnout, impaired professional communication and reduced occupational functioning [14, 16]. Within midwifery, evidence remains comparatively limited, but international research suggests that bullying is embedded within some workplace cultures and may be perpetrated by colleagues, supervisors, managers and medical staff [6, 17–19].

Hierarchical power is particularly evident in clinical education and early‐career practice. Students and newly qualified midwives may be vulnerable because of their limited experience, precarious professional status and dependence on assessment, mentorship and access to clinical learning [20, 21]. Bullying may contribute to reduced confidence and withdrawal from training or the profession, while its persistence despite longstanding recognition suggests that it can become normalised within workplace cultures [17, 22].

The consequences extend beyond individual distress to absenteeism, burnout, turnover and workforce instability [23, 24]. Concerns have also been raised about implications for maternity care. The Ockenden Review [25], for example, identified bullying, fear of speaking up and organisational silence within a maternity service affected by serious clinical failures. However, such evidence supports concern about possible implications rather than establishing a direct causal relationship between bullying and patient outcomes.

Effective responses therefore require more than policies or individual coping strategies. Fear of retaliation, distrust of reporting processes and inadequate organisational follow‐through may discourage reporting and allow harmful conduct to continue [26]. Proposed responses include accessible reporting systems, trauma‐informed support, leadership accountability, respectful workplace cultures and targeted assistance for affected individuals alongside organisational reform [27, 28].

Evidence concerning bullying among midwives in Aotearoa New Zealand remains sparse. International and Australian research indicates substantial exposure, but comparable national evidence is limited, and bullying is shaped by local organisational, cultural and policy contexts [29, 30]. Contextually grounded research is therefore needed to examine how bullying is experienced and how participants connect it with psychological and occupational consequences, retention and concerns about maternity‐care quality. This is particularly important amid significant workforce shortages and resource pressures [31].

## 2. Methodology

### 2.1. Design and Analytic Orientation

The study used a qualitative descriptive design, with inductive thematic analysis followed by theoretically informed ecological interpretation. Initial coding focused primarily on participants’ descriptions and attributed meanings. Later interpretation examined these accounts in their relational, organisational and wider professional contexts. The analysis was informed by a contextualist position: Accounts were understood within their social and organisational settings, while recognising the researchers’ role in constructing meaning. Coding comparison supported reflexive discussion rather than measurement of intercoder agreement.

### 2.2. Participants and Recruitment

Recruitment materials invited registered and student midwives who self‐identified as having experienced workplace bullying within the previous 5 years. They included WorkSafe New Zealand’s [32] definition of bullying as repeated and unreasonable behaviour directed towards a worker or group that can cause physical or psychological harm; this definition was reiterated at interview. Eligibility was determined prospectively through participants’ self‐identification against this definition, without a separate behavioural screening interview. During analysis, repetition or persistence, power asymmetry and difficulty defending oneself were considered as analytic features [1], not retrospectively applied as additional eligibility criteria. Isolated conflict or incivility was distinguished from bullying where the accounts allowed. Harassment, discrimination, racism and cultural marginalisation were treated as potentially intersecting but analytically distinct processes.

Seven practising midwives participated, ranging from their third year of practice to approximately 2 decades or more of experience. Their roles spanned community‐based lead maternity care, primary birthing units, hospital practice, management and other maternity services. This variation supported exploration across practice contexts but did not represent the wider Aotearoa New Zealand midwifery workforce.

Ten Phase I respondents had consented to follow‐up contact and were invited to participate in Phase II. One subsequently participated in a Phase II interview, while the remaining six Phase II participants were recruited independently through advertisements circulated via professional midwifery forums, publications and social media. Although one participant took part in both phases, the interviewers and analysts did not access or link that participant’s individual Phase I responses during Phase II interviewing or analysis. Only aggregate Phase I findings informed the interview guide. Information power was used to consider whether the focused aim, participants’ direct experience and the depth of the interviews could support exploratory thematic interpretation [33]. These features do not establish saturation, comprehensive ecological coverage or representation of the profession. The implications of self‐selection are addressed in the limitations.

Recruitment remained open for approximately 3 months. Although participants could discuss witnessed incidents, personal experience of bullying was required. Ten people expressed interest; seven were interviewed, and three chose not to proceed, without providing reasons. Recruitment ended when the period closed after all eligible and willing respondents had been interviewed. The final sample was therefore pragmatic rather than based on data saturation. No participants withdrew after commencing participation.

Demographic questions were voluntary, and missing information, including employment and geographic details, was not inferred. Characteristics are reported in broad categories to reduce deductive‐identification risk within this small, close‐knit profession (Table 1).

**TABLE 1:** Broad characteristics of participants.

Participant	Midwifery experience	Main practice setting
P1	10–15 years	Hospital‐based practice
P2	10–15 years	Hospital‐based practice
P3	10–15 years	Hospital‐based practice
P4	10–15 years	Hospital‐based practice
P5	Less than 5 years	Primary birthing unit
P6	More than 15 years	Community/LMC practice
P7	More than 15 years	Independent midwifery practice

*Note:* All participants were registered midwives who had personally experienced workplace bullying. Categories have been deliberately broadened to reduce the risk of deductive identification.

### 2.3. Data Collection

Semistructured interviews were conducted online using a guide informed by aggregate Phase I findings concerning authority‐linked bullying, exclusion, false narratives and victim blaming, fear of speaking up, and reported personal and professional consequences. The guide covered the nature and context of bullying, perceived antecedents, professional and personal consequences, coping and responses, reporting experiences and suggested prevention or support. Interviewers used the guide to explore participants’ accounts rather than to confirm the Phase I findings. Interviews were audio‐recorded with consent and transcribed verbatim.

Core research‐team members conducted two interviews, and an experienced qualitative interviewer engaged for the study conducted the remaining five. No interviewer had a prior relationship with participants. The external interviewer had extensive experience conducting sensitive qualitative interviews and received study‐specific briefing and training from the research team. All interviewers used the same semistructured guide and a common probing approach, using broad, open‐ended follow‐up questions to clarify participants’ accounts and explore how experiences unfolded across interpersonal, organisational and professional contexts. The external interviewer debriefed with the research team after each interview. Differences in interviewer style may nevertheless have influenced the depth, emphasis and direction of participants’ accounts. Interviews lasted approximately 60–90 min, yielding an estimated 7–10.5 h of interview data.

### 2.4. Data Analysis

Analysis combined an inductive, data‐led stage with subsequent theoretically informed ecological interpretation. Two researchers repeatedly read and independently coded the transcripts using NVivo. Initial codes reflected participants’ experiences and processes, including public belittling, exclusion, professional undermining, fear, self‐doubt, exit, reporting barriers, leadership inaction, workload pressure and cultural marginalisation. Codes were compared within and across interviews and developed into candidate themes around shared processes rather than assigned immediately to ecological levels. The researchers compared their interpretations and collaboratively reviewed developing patterns, code definitions and theme boundaries. This process was intended to strengthen interpretive depth and reflexivity rather than calculate intercoder agreement or establish a single objectively correct coding framework.

All seven transcripts were reviewed at case level for evidence of repetition or persistence, power imbalance and difficulty defending oneself. All accounts contained evidence consistent with these features. Where participants described challenging or reporting the conduct, difficulty defending oneself was understood as constrained capacity to stop the behaviour or obtain an effective organisational response, rather than an absence of agency.

Candidate themes were reviewed collaboratively for internal coherence, distinction and variation across participants. Attention was given to accounts that qualified the dominant patterns, including one in which accumulated complaints eventually resulted in organisational action. Four themes were constructed: (1) being diminished and pushed out; (2) hierarchy turns vulnerability into control; (3) organisational silence protects harmful practice; and (4) system conditions normalise and isolate.

Only after their development were the themes formally mapped to Johnson’s [8] ecological levels. Initial codes and themes were therefore developed from participants’ accounts rather than assigned to predetermined ecological categories. However, the research question and contextual focus were theoretically sensitised, and this sequencing did not eliminate the possible influence of the Phase I findings or the researchers’ familiarity with the ecological framework.

To support analytic transparency, selected transcript extracts were linked to their corresponding codes, themes and ecological interpretations and checked against their original contexts. Extracts were examined as direct evidence, evidence connecting more than one ecological level, or evidence that qualified the dominant interpretation.

The transcripts were also examined for material that qualified or complicated the ecological interpretation. This included eventual organisational action following sustained persistence, intervention by people receiving care, support from a trusted ‘safe person’, and collective and institutional pressure experienced by a participant occupying a management position. These accounts showed that formal rank did not consistently determine vulnerability and that organisational and relational contexts could operate as sources of either harm or protection. No account was wholly inconsistent with an ecological interpretation, and the themes did not require modification of Johnson’s model. Competing theoretical frameworks were not formally tested.

Evidence was treated as cross‐level when participants explicitly connected experiences or responses occurring at more than one ecological level. Material that could plausibly support more than one interpretation was retained as overlapping rather than treated as evidence of a directional relationship. The proposed pathways were therefore treated as possible patterns suggested within the dataset, with uneven support across participants, rather than as established or universally recurring mechanisms.

### 2.5. Researcher Reflexivity

Both researchers are experienced qualitative researchers who have previously studied workplace bullying. Their backgrounds encompass health, management and the social sciences; neither was a practising midwife. Previous bullying research provided conceptual sensitivity but risked interpretation through established assumptions. Reflexivity was supported through independent coding followed by comparison and discussion. Interpretive differences encouraged alternative readings and examination of how disciplinary backgrounds, prior knowledge and responses to participants’ accounts shaped code and theme development. Reflexive discussion also considered how the external interviewer’s position outside the core research team and differences among interviewers may have shaped data generation. Accordingly, themes are presented as contextualist interpretations constructed through engagement between participants’ accounts, the researchers and the ecological framework, rather than as objective categories discovered independently of the research process.

### 2.6. Ethics and Participant Well‐Being

Ethical approval was obtained from the relevant university human ethics committee. Before interviewing, participants received study information and provided written consent to participate, be audio‐recorded and have de‐identified quotations reported. Participation was voluntary: Participants could stop the interview, discontinue recording and determine whether material discussed after recording ceased could be used. Identifying details were removed or generalised because midwifery is a small professional community.

The external interviewer transferred recordings and other interview materials to the lead researcher, who stored them with transcripts and associated files in password‐protected institutional cloud storage. Access to identifiable recordings and the participant key was restricted to the lead researcher. The second analyst accessed de‐identified transcripts for independent coding through a restricted institutional cloud‐storage folder.

The participant information sheet advised that the researchers could not provide advice about participants’ situations and identified sources of professional and community support, including a trusted colleague, union representation, workplace EAP services, Lifeline and Samaritans. Participants were informed that they could stop at any time, request that recording cease and resume the interview only if they wished. When participants became distressed during interviews, interviewers offered to pause and checked their willingness to continue. One participant retrospectively described a period of suicidality associated with her workplace experience. The interviewer offered to pause and subsequently asked whether she had accessed therapeutic support. The participant chose to continue, reported having obtained psychological support and described herself as now being in a stronger position. No current suicidal intent was expressed during the interview. The study did not involve formal clinical risk assessment, and no referral, escalation or post‐interview follow‐up was documented.

### 2.7. Findings

The analysis generated four process‐oriented themes from participants’ accounts before interpretation through the ecological model. The themes connect participants’ descriptions of interpersonal experiences with their accounts of organisational responses and wider professional conditions. The theme labels are interpretive constructions, not verified causal mechanisms. The four themes are detailed below, with illustrative quotations attributed using participant identifiers P1–P7.

#### 2.7.1. Theme 1: Being Diminished and Pushed out

Participants described an accumulating process of public diminishment, professional undermining and progressive withdrawal. Interpersonal conduct extended into fear, self‐doubt, psychological distress and erosion of professional identity. P6 recalled public denigration during training that was countered only when the people receiving care intervened:

“The midwife proceeded to tell them (patients), in front of me, how useless I was, because I couldn′t do something, and she proceeded to tell them how utterly useless I was. You know, belittled in front of women.” (P6)

This incident linked relational humiliation with a status hierarchy in which inexperience discredited the learner. Its effects extended beyond the encounter. P1 said that mistreatment ‘*made me question my worth as a midwife’* and whether they should continue practising. P2 recounted a period of severe distress and suicidality associated with the workplace experience:

“When I saw her car in the car park, I’d get really anxious that she was coming in, that I was going to have to deal with her. My stress levels went through the roof… I got to the point where I was suicidal about it. I didn’t want to work anymore. I didn’t want to be a midwife anymore. The only way I could think of to make it all go away was by taking myself out of the equation.” (P2)

P7 reached ‘*the periphery of a complete breakdown’* and felt that ‘*they make us feel worthless’,* while P3 sought counselling to determine ‘*what was wrong with me’* after repeated complaints. Withdrawal was therefore not simply an individual coping preference: P2 used an injury to ‘*escape’* a workplace, P3 resigned from management, and P5 considered leaving valued work because continually watching their own back was ‘*not worth [their] mental health’.* Participants also used avoidance, psychological support, reflective writing and trusted colleagues or family. Collectively, sustained and insufficiently addressed interpersonal devaluation became internalised as professional self‐doubt and made exit appear protective.

#### 2.7.2. Theme 2: Hierarchy Turns Vulnerability Into Control

Participants associated vulnerability to bullying with professional status and relational power that allowed some people to define others as incompetent, risky or out of place. Early‐career and student status were prominent vulnerabilities. P5 entered a role amid open opposition and later learnt that a colleague had anticipated their failure:

“Not one person congratulated me for getting the job. All I could hear was that [another person] should have got the job; everyone was quite happy to say that to my face. Three years later, a midwife said to me, ‘When you got the job, I was just waiting for you to [mess] up every single shift… so that I could say… you shouldn’t have put a new grad in a position like that.’” (P5)

P5 described new‐graduate status as becoming a basis for surveillance and presumed incompetence. P2 was ‘absolutely terrified of stepping out of my lane as a student midwife’ because doctors would ‘*hound students*’*.* P6 interpreted the treatment of capable students as an attempt by insecure senior practitioners to ‘*put [them] down a peg or two’.* P2 also described a perpetrator warning doctors and senior midwives that the participant required monitoring.

Hierarchy also operated through control over access to work:

“She said, ‘I can’t guarantee you any hours,’ and I felt that comment was, ‘I’m in a position of power above you. If you go casual, I can’t guarantee you any hours,’ when I knew fully well they were so short‐staffed they would have work coming out of my ears.” (P1)

P1 interpreted insecure hours as providing practical leverage despite reported staff shortages. Similarly, recruiting doctors and senior midwives to monitor P2 converted one person’s judgement into a wider reputational constraint. P4 extended this pattern to interprofessional and intraprofessional boundaries, describing doctors who worked with someone for years without acknowledging them and maternity roles treated as ‘*persona non grata’.* Hierarchy therefore gave informal judgements practical force by shaping credibility, surveillance, work access and the perceived safety of speaking or learning.

#### 2.7.3. Theme 3: Organisational Silence Protects Harmful Practice

Participants described reporting systems that demanded visible evidence of conduct that was often covert, cumulative or unwitnessed. P1 anticipated that without documentary proof, ‘*nothing’s going to happen’.* Organisational responses were often perceived as containing complaints rather than addressing behaviour. P2 described a collective complaint and attempted escalation:

“Our unit ended up putting in a big complaint about it. It went nowhere, and it got quashed by management. But I got over my anxiety and I got angry, and I actually went and had a meeting with the CEO about how upset I was about what had happened. That obviously went nowhere either.” (P2)

P2 described formal escalation as failing to provide redress. P1 described an incident being treated as though it had never happened: *“This will be our little secret.”* Observing another case led P5 to conclude, *“I’m not safe here. I could never complain… because look at how that was dealt with.”* Organisational handling therefore influenced both complainants and other workers’ willingness to speak.

The pattern was not absolute. P4 described eventual action after complaints accumulated:

“There was one particular labour ward coordinator who was well known and had a huge file of complaints against her, but the managers wouldn’t get rid of her because she was a good midwife. She used to make midwives cry… Eventually she was dismissed. There were enough complaints against her.” (P4)

P4’s account qualifies the pattern of perceived inaction and suggests that action was possible in that case after complaints accumulated; it also illustrates the evidentiary threshold this participant believed was required. Participants interpreted organisational responses as signalling what workers might expect if they spoke; in their accounts, silence was associated with high evidentiary thresholds, contained or inconclusive investigations, limited follow‐through and expectations that reporting would be ineffective or unsafe.

#### 2.7.4. Theme 4: System Conditions Normalise and Isolate

Participants situated bullying within wider workforce, employment, and professional and cultural arrangements. Participants associated staffing shortages and workload pressure with strained relationships, reduced support and heightened perceived consequences of mistakes. P6 contrasted public reassurance from leadership with conditions inside an understaffed service:

“Things are in dire straits. There’s not enough staff to look after people, midwives are in short demand, and there’s such a shortage. People are terrified something terrible’s going to happen most days when they go to work. They have to work so hard; they’re so burnt out, they’re so stressed out… You can tell [leadership] there’s a problem, and they don’t try and fix it.” (P6)

P5 likewise worked at night while ‘*terrified that something bad’* would happen because there was no one to call. These accounts do not establish that resource constraints caused bullying; they show how scarcity made harmful conduct harder to challenge and organisational reassurance appear disconnected from everyday conditions.

Employment arrangements deepened isolation. P7 stated, ‘*You are in a self-employed capacity where you’ve got no support at all’,* while P6 described community‐based midwives working outside hospitals, being paid differently and navigating a ‘*tricky’* payment schedule. Macrolevel funding and employment design were thus experienced through limited organisational protection and individual vulnerability.

Two participants described cultural marginalisation intersecting with workplace hostility. P3 connected repeated complaints with resistance to te reo Māori and culturally appropriate practice:

“They liked me when I stayed in [another community]. They did not like the fact that I could speak te reo Māori. They did not like the fact that I brought culturally appropriate things into our unit. One of the first complaints was for bullying and my exclusionary behaviour because I spoke Māori in the hospital and I must be made to stop.” (P3)

P5 similarly described Te Tiriti education as being framed as ‘*them and us’* within their class. These bounded accounts do not establish the experiences of Māori midwives or the operation of the bicultural health system generally. They indicate that bullying, racism and cultural marginalisation may intersect without being analytically collapsed.

Conformity was also portrayed as protective, and speaking out as dangerous. P7 stated that those who did not ‘stay within [their] lane’ could be made a victim, while P2 remained with a ‘*safe person’* to complete training because bullying seemed to be ‘*all around’.* Together, these accounts connect macrolevel workforce, employment, professional and cultural conditions with organisational support, interpersonal conduct and individual self‐protection.

#### 2.7.5. How the Ecological Levels Interact

The accounts suggested two possible cross‐level connections, summarised in Table 2. First, some participants connected workforce scarcity or limited immediate support with pressured working environments, perceived leadership inaction, interpersonal undermining and fear or withdrawal. P6 provided the clearest account of workforce pressure and leadership response; P1 referred to staffing when discussing insecure hours, and P5 described limited immediate night support. The full sequence was not demonstrated in every account. Second, participants connected employment and professional‐status arrangements with uneven access to organisational protection, exclusion, surveillance, withholding of support and isolation. These connections are an interpretive synthesis that generates hypotheses rather than causal relationships established across Aotearoa New Zealand midwifery.

**TABLE 2:** Data‐derived themes and their ecological interpretation.

Data‐derived theme	Ecological levels referenced	Proposed interpretive connection
Being diminished and pushed out	Meso and micro	Interpersonal devaluation becomes psychological harm and withdrawal.
Hierarchy turns vulnerability into control	Macro, meso and micro	Professional status enables surveillance, silencing and constrained learning.
Organisational silence protects harmful practice	Exo, meso and micro	Reporting barriers and inaction legitimise conduct and discourage future voice.
System conditions normalise and isolate	Macro, exo, meso and micro	Workforce, employment and cultural arrangements shape support, interaction and vulnerability.

## 3. Discussion

This study identified four processes through which seven midwives understood workplace bullying across ecological levels. The pathways summarised in Table 2 show how, within participants’ accounts, interpersonal conduct gained force through professional hierarchy, organisational responses and wider employment, workforce and cultural conditions. These findings do not suggest that bullying operates uniformly across Aotearoa New Zealand midwifery but move beyond categorising examples by ecological level towards examining connections among them.

### 3.1. Being Diminished and Pushed out

Public belittling, exclusion and professional undermining accumulated into anticipatory anxiety, self‐questioning, psychological distress and withdrawal, consistent with established psychological and occupational consequences of bullying [14, 34]. Participants added process detail by describing how attacks on professional legitimacy made remaining in a role untenable and an exit a form of self‐protection rather than individual failure to cope.

Participants described exhaustion alongside sustained workload pressure, complaint processes and inadequate support. This is consistent with wider evidence that nursing burnout can arise from continuing work‐related stressors rather than a single crisis [35]. Burnout was not separately assessed in the present study; participants’ reported exhaustion is therefore treated as a consequence or contextual process, not as a demonstrated cause of bullying. Responses focused solely on resilience or individual coping may leave unchanged the conditions participants associated with depletion.

### 3.2. Hierarchy Turns Vulnerability Into Control

Hierarchy made judgements about competence consequential. Participants associated student, new‐graduate and less powerful professional positions with intensified scrutiny, restricted learning and control over who could speak with authority. This accords with evidence that hierarchical clinical environments facilitate bullying where assessment, access to work or professional reputation depends on senior colleagues [3, 21]. Hierarchy also extended beyond formal rank to status differences between medicine and midwifery and within the maternity workforce.

Such internally embedded harm may be less visible than external violent incidents. Cao et al.‘s [36] analysis of Chinese social media and news accounts showed that representations of workplace violence vary across platforms and discursive layers. Although not directly transferable to Aotearoa New Zealand midwifery, it helps distinguish highly visible external violence from the subtle, repeated and professionally mediated conduct described here.

### 3.3. Organisational Silence Protects Harmful Practice

Participants described harmful conduct as persisting where, in their experience, reporting systems required discrete, witnessable evidence, whereas many behaviours were covert, cumulative or reputational. Unsubstantiated or apparently contained complaints were consequently interpreted as organisational tolerance, consistent with evidence that speaking‐up systems are weakened by anticipated retaliation, doubtful follow‐through and difficulty translating relational harm into an admissible incident [26].

Organisational inaction was not absolute: One participant described a perpetrator being dismissed after complaints accumulated. This discrepant account indicates that accountability was possible in the organisation described by P4, while showing variation in participants’ reported organisational responses.

### 3.4. System Conditions Normalise and Isolate

Participants situated bullying within workforce scarcity, employment arrangements, professional norms and cultural dynamics. They associated staffing shortages and workload pressure with intensified stress and reduced support and described self‐employment and contracting as contributing to isolation and uncertain access to organisational protection. These are participants’ interpretations of plausible contextual conditions rather than evidence that under‐resourcing or employment design caused bullying.

Two participants described resistance to te reo Māori, culturally appropriate practice or the teaching of Te Tiriti. Their accounts may reflect intersecting bullying, racism, discrimination and cultural marginalisation, but these constructs are not interchangeable. This was not Kaupapa Māori research and cannot characterise Māori midwives, racism across the profession, bicultural healthcare relations or Te Tiriti implementation. Interpreting these accounts solely through a bullying framework could obscure distinct discriminatory processes. Further investigation should use Māori‐led or culturally grounded methodologies.

Participants also raised gendered devaluation through professional discourse and midwifery’s positioning within medicine. References to a ‘mean girls’ culture are insufficient to establish a gender‐based macrolevel explanation; gendered expectations are therefore treated as tentative contextual propositions requiring further investigation rather than demonstrated findings.

Taken together, participants’ accounts suggest possible connections among individual conduct, relational power and structural context across ecological levels. Figure 2 presents two possible cross‐level connections suggested across participants’ accounts without treating structural conditions as solely responsible or the ecological levels as predetermined thematic categories.

**FIGURE 2:**
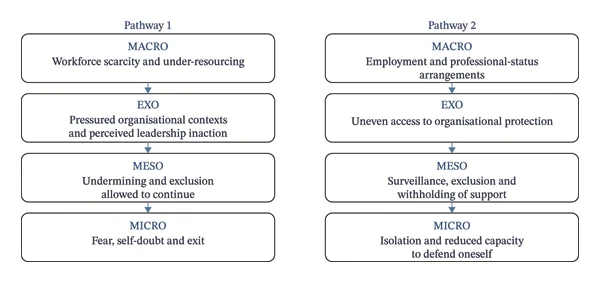
Possible cross‐level connections suggested across participants’ accounts. Note: Connections are an interpretive synthesis; they are not established causal effects or sequences demonstrated in every account.

### 3.5. Implications for Practice, Organisations and Policy

Participants’ accounts suggest candidate intervention targets for further investigation across relational, organisational and wider professional levels. Table 3 distinguishes needs grounded in participants’ accounts from responses informed by existing literature. None of the proposed responses was evaluated in this study, and their effectiveness remains untested.

**TABLE 3:** Candidate intervention targets generated from participants’ accounts and ecological interpretation.

Level	Finding‐based problem	Candidate intervention target	Caution	Basis/status
Micro	Psychological distress, self‐doubt and withdrawal	Confidential, trauma‐informed support; timely access to clinical supervision and independent assistance.	Support should not substitute for action on relational and structural conditions.	Participant‐grounded need; response literature‐informed; not evaluated.
Meso	Belittling, exclusion, targeting and hierarchy	Behaviourally explicit expectations; preceptor/supervisor development; active‐bystander and peer‐support practices.	Training alone is unlikely to change conduct without accountability.	Participant‐grounded need; response literature‐informed; not evaluated.
Exo	Reporting barriers, leadership inaction and organisational silence	Accessible reporting pathways; antiretaliation safeguards; independent review; documented follow‐through and monitoring of recurrent patterns.	Systems must recognise cumulative and covert conduct while preserving procedural fairness.	Participant‐grounded reporting problem; response literature‐informed; not evaluated.
Macro	Employment isolation, workforce pressure and professional/cultural norms	Review how contracting, funding and professional accountability affect access to support; address workforce resourcing; investigate culturally marginalising practices through Māori‐led or culturally grounded research.	The study identifies issues for investigation; it does not establish policy effectiveness or nationwide prevalence.	Exploratory investigation target; not an evaluated policy recommendation

Patient and maternity‐care safety was not directly examined. Participants sometimes linked diminished confidence, pressured work and relational hostility to concerns about care, but these accounts cannot establish an effect on clinical outcomes. Any possible implications for safer care are contextual and literature‐based and are not outcomes demonstrated by this study. Similarly, national retention strategies and policy reform are potential applications requiring further investigation, rather than conclusions established by this exploratory study.

## 4. Conclusion and Limitations

This exploratory study illustrates how seven participating midwives connected interpersonal mistreatment with organisational responses and wider professional conditions. The data‐derived themes and ecological interpretation make these perceived connections visible without establishing profession‐wide causal mechanisms. The framework generated empirically informed propositions and candidate intervention targets rather than demonstrating how bullying operates across Aotearoa New Zealand midwifery.

The findings should be interpreted in light of the study’s small, self‐selected sample and reliance on retrospective self‐report. Recruitment specifically sought people willing to identify and narrate workplace bullying and may therefore have preferentially attracted experiences that remained particularly severe, unresolved or salient. The design cannot compare how different workers interpret similar encounters or distinguish experiences that escalated into perceived bullying from difficult workplace relationships that did not. The study did not include the perspectives of managers, human‐resource personnel, regulators, alleged perpetrators, nontarget witnesses or people receiving maternity care. Consequently, participants’ descriptions of organisational processes and responses could not be independently verified. Its limited contextual and demographic range restricts transferability, and the sample cannot provide comprehensive coverage of ecological mechanisms across the profession or support national generalisation. Interpreting cultural marginalisation solely through a bullying framework may also obscure distinct experiences of racism or discrimination.

Researcher positioning, theoretical sensitisation and differences in interviewer interaction may also have shaped data generation and interpretation. Although the ecological model was formally applied after inductive coding and theme development, familiarity with the model and the Phase I findings may have drawn attention towards material corresponding with its levels. Competing theoretical frameworks were not formally tested. Distinctions among bullying, conflict, incivility, harassment, racism and discrimination were not always clear, and the limited cultural accounts were not interpreted using a Māori‐specific methodology.

Taken together, participants’ accounts suggest possible connections among interpersonal mistreatment, professional hierarchy, organisational responses and wider professional or structural conditions. The proposed cross‐level pathways are empirically informed interpretations requiring further examination rather than established processes operating across the profession. The ecological analysis identifies candidate intervention points rather than solutions evaluated by this study. Future research should examine these propositions using larger and more diverse samples, incorporate organisational, regulatory and other stakeholder perspectives and evaluate multilevel responses extending beyond individual coping.

## Funding

This research received no specific grant from any funding agency in the public, commercial or not‐for‐profit sectors.

Open access publishing facilitated by Massey University, as part of the Wiley ‐ Massey University agreement via the Council of Australasian University Librarians.

## Disclosure

This project was performed as part of the employment of the authors at Massey University.

## Ethics Statement

This project has been reviewed and approved by the Massey University Human Ethics Ohu Matatika 2, Application OM2 25/17. If you have any concerns about the conduct of this research, please contact the Chairperson, Massey University Human Ethics Ohu Matatika 2, email humanethics2@massey.ac.nz.

## Conflicts of Interest

The authors declare no conflicts of interest.

## Data Availability

The data that support the findings of this study are not publicly available due to ethical and privacy restrictions associated with the sensitive nature of the qualitative interview data and the potential risk of participant identification.
